# Anopheline mosquitoes of north-western Russia (Diptera, Culicidae): updated distribution and morphological characters

**DOI:** 10.3897/BDJ.13.e164756

**Published:** 2025-08-27

**Authors:** Sergey V. Aibulatov, Daria I. Lebedeva, Alexei V. Khalin, Natalia A. Lyutikova, Daniil D. Fedorov, Liubov A. Bespyatova, Alexei V. Polevoi, Gelena A. Lunina, Sergey V. Bugmyrin

**Affiliations:** 1 Institute of Biology of the Karelian Research Centre of the Russian Academy of Sciences, Petrozavodsk, Russia Institute of Biology of the Karelian Research Centre of the Russian Academy of Sciences Petrozavodsk Russia; 2 Zoological Institute, Russian Academy of Sciences, St. Petersburg, Russia Zoological Institute, Russian Academy of Sciences St. Petersburg Russia; 3 Forest Research Institute of the Karelian Research Centre of the Russian Academy of Sciences, Petrozavodsk, Russia Forest Research Institute of the Karelian Research Centre of the Russian Academy of Sciences Petrozavodsk Russia; 4 Saint-Petersburg Pasteur Institute, St. Petersburg, Russia Saint-Petersburg Pasteur Institute St. Petersburg Russia

**Keywords:** *Anopheles maculipennis*, morphometry, ITS2, palpomere, flagellomere

## Abstract

Mosquitoes of the genus *Anopheles* were collected in the Republic of Karelia, St. Petersburg, Leningrad Region, Novgorod Region and Pskov Region (Russia) in order to clarify their distribution and genetic and morphological diversity. ITS2 sequence analysis of *An. maculipennis* s. l. (58 specimens) showed that they belonged to *An. beklemishevi*, *An. messeae*, *An. daciae* and *An. maculipennis* s. str. Taking into account our earlier data on *An. claviger*, this means that there are five *Anopheles* spp. in north-western Russia whose records are confirmed by molecular methods. We revealed *An. daciae* in Leningrad and Pskov Regions for the first time and confirmed its distribution in Karelia. *Anopheles beklemishevi* was found near the Arctic Circle in north-western Karelia. The study of morphology of the female heads of *An. daciae*, *An. messeae* and *An. beklemishevi* showed they may potentially be used for differentiation of these species.

## Introduction

Mosquitoes of the subfamily Anophelinae (Diptera, Culicidae) or ‘anophelines’ have a worldwide distribution. Anopheline females feed on mammals and/or birds, with species readily attacking humans. Some mosquitoes of the genus *Anopheles* Meigen, 1818 transmit malaria parasites, microfilariae and arboviruses to humans and other animals. *Anopheles maculipennis* Meigen, 1818 is associated with various viruses, such as Batai, Babanki, Calovo, Marburg, Myxoma, Rift Valley fever, Western equine encephalomyelitis and West Nile virus (Wilkerson et al. 2021), as well as *Plasmodium vivax* (Grassi & Feletti, 1890). Moreover, Sindbis virus was isolated from *An. maculipennis* s. l. in Fennoscandia (Wilkman et al. 2023). *Anopheles messeae* Falleroni, 1926, *An. maculipennis* s. str. and, probably, *An. daciae* Linton, Nicolescu & Harbach, 2004 are responsible for malaria transmission in some parts of Europe where their incidence is high (Becker et al. 2020).

**Figure 1.**
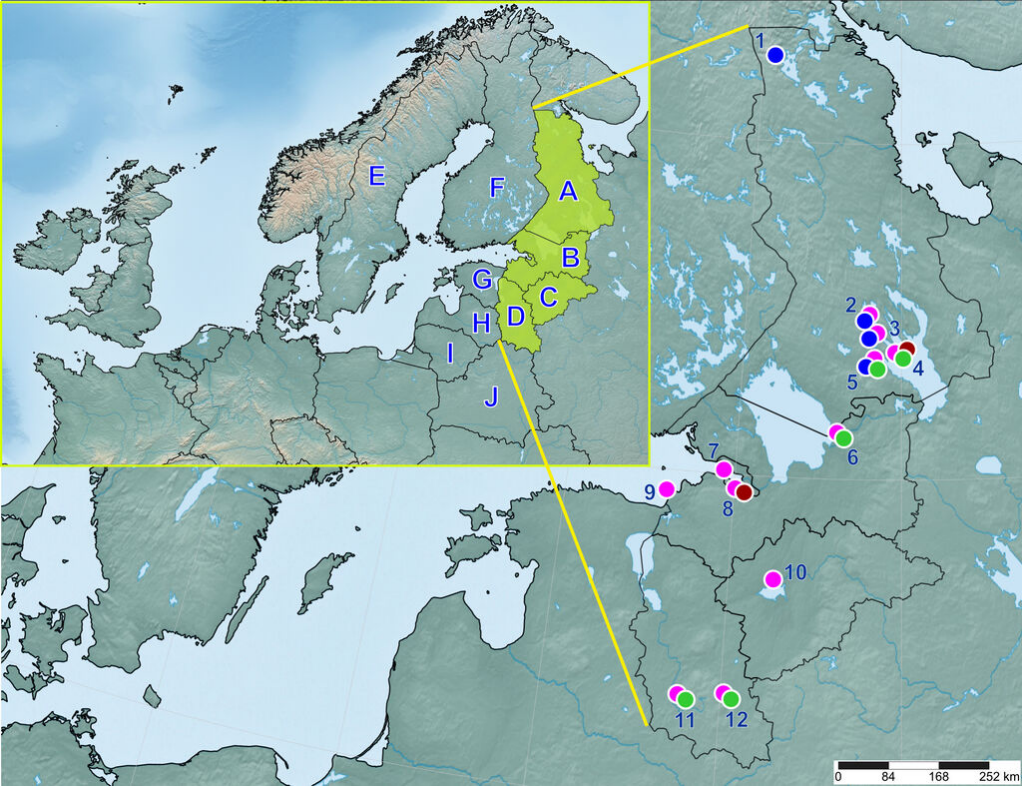
**Collection localities of anopheline mosquitoes in north-western Russia**. Designations: blue circles – *Anopheles beklemishevi*; magenta circles – *Anopheles messeae*; green circles – *Anopheles daciae*; brown circles – *Anopheles maculipennis* s. str. The insert shows north-western Russia on a map of northern Europe. North-western Russia. *A* – Karelia; *B* – St. Petersburg and Leningrad Region; *C* – Novgorod Region; *D* – Pskov Region. Other countries. *E* – Sweden; *F* – Finland; *G* – Estonia; *H* – Latvia; *I* – Lithuania; *J* – Republic of Belarus.

North-western Russia (NWR) includes Karelia (Republic of Karelia), St. Petersburg (federal city), Leningrad Region, Novgorod Region and Pskov Region (Fig. 1). NWR is located in northern Europe between 55°N and the Arctic Circle and covers an area of more than 360,000 km^2^. There are several large waterbodies in the area, such as lakes Ladoga, Onega, Peipus and Ilmen, as well as the White Sea and the Baltic Sea. The terrain of NWR displays a gentle hilly relief, the altitudes ranging from a few to hundreds of metres above sea level. The main biotic components of landscapes are coniferous and mixed forests. The climatic system of NWR is a combination of marine and continental climates (Isachenko et al. 1965).

Over five hundred species of anopheline mosquitoes are recorded in the world fauna (Harbach 2025). Only *Anopheles claviger* (Meigen, 1804) and *An. maculipennis* s. l. occur in NWR (Khalin et al. 2021a); *An. claviger* is widespread in Leningrad, Novgorod and Pskov Regions and was recorded in southern Karelia (Khalin et al. 2024).

*Anopheles maculipennis* s. l. or Maculipennis group includes 24 species separated into three subgroups (Harbach 2004; Harbach 2013; Wilkerson et al. 2021). Maculipennis subgroup consists of eight species distributed throughout the Palaearctic, while the Quadrimaculatus subgroup comprises six species recorded in the Palaearctic and the Nearctic and Freeborni subgroup includes four Nearctic species. Three species from the Maculipennis subgroup (*An. maculipennis*, *An. daciae* and *An. messeae*) and one species from the Quadrimaculatus subgroup (*An. beklemishevi* Stegnii & Kabanova, 1976) occur in the northern Palaearctic and in NWR. The northern range of *An. maculipennis* s. str. runs through southern Sweden (Lilja et al. 2020) and Finland (Culverwell et al. 2021) as well as Russia: Karelia (Perevozkin et al. 2012; Moskaev et al. 2024) and Yaroslavl and Chelyabinsk Regions (Novikov and Vaulin 2014) (Suppl. material 1), this species being widespread in the Western Palaearctic. *Anopheles daciae* is found almost throughout Europe from the United Kingdom to Poland, as well as in Russia and Kazakhstan (Calzolari et al. 2021; Smitz et al. 2021; Wilkerson et al. 2021; Ibáñez-Justicia et al. 2022; Brusentsov et al. 2023), its range also passing through Sweden, Finland (Lilja et al. 2020; Culverwell et al. 2021) and Russia: Leningrad and Arkhangelsk Regions and Krasnoyarsk Krai (Brusentsov et al. 2023). *Anopheles messeae* and *An. beklemishevi* are distributed from Western Europe to Yakutia (Russia) and occur as far north as Sweden, Finland (Jaenson et al. 1986; Culverwell et al. 2021) and Russia: Karelia, Komi Republic, Krasnoyarsk Krai and Yakutia (Novikov 2016; Brusentsov et al. 2023; Moskaev et al. 2024).

*Anopheles claviger* is a member of Claviger complex, which comprises two species distributed in the Western Palaearctic (Harbach 2004; Becker et al. 2020). It differs from *An. maculipennis* s. l. by the absence of spots formed by dark scales on the wings of adults and branching of the outer clypeal seta (3-C) in the larva. However, both adults and larvae of *An. maculipennis* s. str., *An. daciae*, *An. messeae* and *An. beklemishevi* are morphologically similar and require new morphological and molecular approaches for the identification of these species (Becker et al. 2020).

Since morphological identification of some species of the Maculipennis group is challenging, they are distinguished with the use of molecular methods. However, *Anopheles daciae* and *An. messeae* were not recognised as two separate species after application of DNA markers, such as mitochondrial cytochrome c oxidase I (COI), NADH dehydrogenase subunit 4 gene (ND4) and NADH dehydrogenase subunit 5 gene (ND5). The authors concluded that more evidence was necessary to support the hypothesis that *An. daciae* and *An. messeae* were distinct species (Lilja et al. 2020; Smitz et al. 2021; Calzolari et al. 2024). The only markers currently considered useful for identifying the species of Maculipennis group are nuclear internal transcribed spacer 2 (ITS2) and the characters of polytene chromosomes (see, for example, Linton et al. (2003); Nicolescu et al. (2007); Kampen (2005); Artemov et al. (2018), Artemov et al. (2021); Calzolari et al. (2021); Smitz et al. (2021); Moskaev et al. (2024)).

In this paper, we present new data on the distribution of *Anopheles maculipennis* s. str., *An. daciae*, *An. messeae* and *An. beklemishevi* based on ITS2. We also assess the potential applicability of morphological characters of the female head for identification of the species within *An. maculipennis* s. l., with the focus on the length of palpomeres and flagellomeres as recommended by Gutsevich (1972), Gutsevich (1973a) and Gutsevich (1973b).

## Material and methods

**Mosquito sampling**

The specimens were collected from January to November 2022–2024 in twelve collection localities in Karelia, St Petersburg, Leningrad, Novgorod and Pskov Regions (Fig. 1, Suppl. material 1, Suppl. material 2, Suppl. material 3). We used standard techniques for sampling mosquitoes and rearing of immatures (Khalin et al. 2021b). Resting adults and hibernating females were collected with a chamber aspirator, while flying females were collected with a Krystal trap or a Mosquito Magnet® trap (Pioneer design, Octenol as attractant). The sampling of Mosquito Magnet trap continued for one full day or a shorter period from May to September, traps being examined at 2-h intervals. Larvae were sampled with a dipper, reared in wide 500-ml containers and fed on powdered fish food. We used SimpleMappr (Shorthouse 2010) to generate a template for the maps (Fig. 1, Suppl. material 1). The following are the collection points, their coordinates and the sampling techniques.

Karelia

1. Paanajärvi National Park. 66.243°N; 30.5639°E. Meadow near the Olonga River, mixed forest. Attacking females were sampled with a Mosquito Magnet® trap at 26–29.06.2024 (Suppl. material 2, A).

2. Village of Munozero, shed. 62.2521°N; 33.8244°E. Pine and mixed forests. Hibernating females were sampled on the walls of the shed at 04.04.2024.

3. Village of Gomselga. Adults: Research station of IB KarRC RAS (Institute of Biology of the Karelian Research Centre of the Russian Academy of Sciences). 62.0683°N; 33.9592°E. Meadow near mixed forest. Attacking females were sampled with a Mosquito Magnet® trap on 17.07–21.08.2024 (Suppl. material 2, B). Larvae: The backwater of Lake Maloe Lindolampi, near the locality where adults were collected, on 01.08.2024 (Suppl. material 2, C). The backwater of Sporki River, near the locality where adults were collected, on 24.08.2023.

4. Village of Pinguba, summerhouse. 61.8679°N; 34.5527°E. Pine forest. Males were sampled with window trap set at the edge of garden plot, on 22.09–13.10.2024.

5. Village of Lososinnoe, an abandoned holiday camp. 61.6701°N; 34.1696°E. Pine forest. Hibernating females were sampled on the walls of a brick house at 27.10.2024.

St. Petersburg and Leningrad Region

6. Area of Gumbaritsy. 60.6764°N; 32.9412°E. Pine forest. Adults: shed. Resting females were sampled on the walls of the shed on 16.08.2023. Larvae: artificial waterbodies, near the locality where adults were collected, on 20.08.2023.

7. Kotlin Island, uninhabited concrete constructions. 60.0259°N; 29.6743°E. Deciduous forest. Resting females were sampled on the ceiling of buildings on 24.09.2024.

8. Volodarsky Garage Cooperative. 59.8217°N; 30.1045°E. Hibernating females were sampled on the ceiling of a concrete garage on 11.10.2024.

9. Kurgalsky State Nature Reserve, abandoned buildings. 59.7764°N; 28.1097°E. Mixed forest. Hibernating females were sampled on the ceiling and the walls of concrete buildings on 25.03.2024.

Novgorod Region

10. Veliky Novgorod, Area of Vitoslavlitsy. 58.4904°N; 31.2726°E. Park with deciduous trees and meadows. Flying adults were sampled on 01.11.2023 (Suppl. material 2, D).

Pskov Region

11. Village of Mochalkovo, farmhouse. 56.5311°N; 28.7979°E. Deciduous forest. Hibernating females were sampled on the walls of a house on 10.01.2022 and 07.10.2023 (Suppl. material 2, E).

12. Village of Bashovo. 56.6554°N; 30.1772°E. Deciduous forest. Adults: shed. Resting females were sampled on the walls of the shed on 27.09.2023 and 18.09.2024 (Suppl. material 2, F). Larvae: A pond with aquatic plants, near the locality where adults were collected, on 30.07.2024.

The materials examined are deposited in the ZIN RAS (Zoological Institute of the Russian Academy of Sciences, St. Petersburg).

The studies were carried out using the equipment (Olympus SZX7, BX57, Leica DM5000B) of the Laboratory for the Study of Parasitic Arthropods (ZIN RAS) and the Core Facility of KarRC RAS (Petrozavodsk).

**Morphological methods**

**Figure 2.**
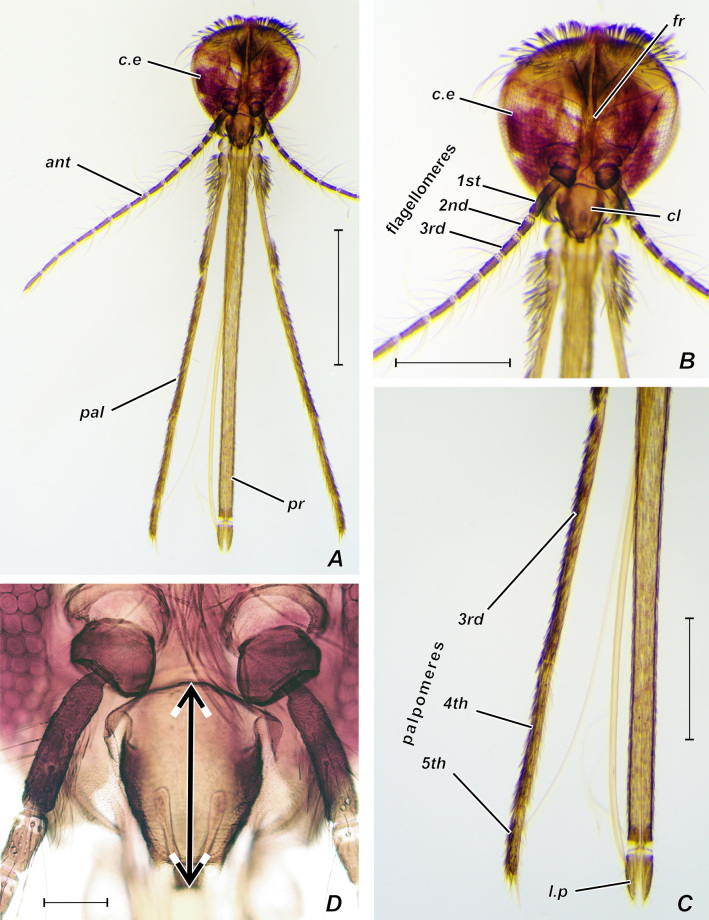
***Anopheles messeae* (*A–C*) and *An. daciae* (*D*), female head, Euparal slide, frontal view**. Designations: *ant* – antenna; *c.e* – compound eye; *cl* – clypeus; *l.p* – labial palps; *fr* – frons; *pal* – maxillary palp; *pr* – proboscis. The arrows show the length of clypeus. Scale bars: 1 mm (A), 0.5 mm (B, C) and 0.1 mm (D).

Mosquito adults and larvae were morphologically identified using the keys of Becker et al. (2020) and additional keys based on the head characters (Gutsevich 1972; Gutsevich 1973a; Gutsevich 1973b). We heated the female heads below the boiling point in a 10% solution of potassium hydroxide for ten minutes and the specimens were mounted on slides using Euparal, following the technique of Khalin and Aibulatov (2024). The head morphology was examined in 49 females. We measured the length of eight structures (Fig. 2): 1^st^, 2^nd^ and 3^rd^ flagellomeres, clypeus, 3^rd^, 4^th^ and 5^th^ palpomeres and labial palps. In case of palpomeres and flagellomeres, the mean between the left one and the right one was calculated.

**Statistical processing of the data**

We performed the multivariate analysis of variance (MANOVA) in order to identify statistically significant differences between species of *Anopheles maculipennis* s. l. for the set of characters. After that, linear discriminant analysis (LDA) was implemented in order to construct a model classifying the species. Data analysis and visualisation of the results were performed in Past 5.1 (Hammer et al. 2001) and R environment (R Core Team 2024) using the packages “car” (MANOVA; Fox and Weisberg (2019)), “MASS” (LDA; Venables and Ripley (2002)) and “ggplot2” (visualisation; Wickham (2016)). A ten-fold cross-validation procedure in the “caret” package (Kuhn 2008) was used for a reliable assessment of the generalisation ability of LDA model.

**Molecular identification**

In total, 58 specimens were examined using ITS2 rDNA ribosomal marker. Genomic DNA was isolated from legs of individual ethanol-fixed specimens using DNAExtran kits (Synthol, Moscow, Russia). Polymerase chain reaction (PCR) for specific amplification of the fragment was conducted using primers CP16 (5^'^-GCGGGTACCATGCTTAAATTTAGGGGGTA-3') and CP17 (5'-GCGCCGCGGTGTGAACTGCAGGACACATG-3') according to Porter and Collins (1991).

PCR products were purified using the Cleanup Standard Extraction Kit (Evrogen, Moscow, Russia) following the manufacturer’s instructions and then sequenced directly with the automatic sequencing system ABI PRISM 3100-Avant (Applied Biosystems Inc., Foster City, CA, USA). All the samples were sequenced with both forward and reverse primers to conﬁrm the presence of single-nucleotide polymorphisms (SNPs) on both DNA strands. Sequenograms were checked in DNA Chromatogram Explorer implemented within the DNA Baser (2013). Consensus sequences were assembled in MEGA v.12 (Kumar et al. 2024).

The identity of the newly-generated sequences was checked with the Basic Local Alignment Search Tool (NCBI BLASTn (1990)) and deposited in GenBank with accession numbers PQ896902-PQ896903 for *Anopheles maculipennis*, PQ897276-PQ897301 for *An. messeae*, PQ897303-PQ897313 for *An. daciae* and PQ899463-PQ899481 for *An. beklemishevi*.

Then the novel sequences of ITS2 region were aligned with those of representatives of Maculipennis group in MEGA v.12 to access the phylogenetic positions of the species. The full list of sequences used for phylogenetic analysis is presented in Suppl. material 3. ITS2 sequence of *Anopheles claviger* (Meigen, 1804) (PP358790) was used as an outgroup (Khalin et al. 2024).

To test the topology of the tree, Maximum Likelihood (ML) analysis with 1000 replicates was investigated by using the best fit ML model calculated in MEGA v.12. This was the Kimura 2-parameter model including the estimates of amongst-site rate heterogeneity and insertions (K2+G+I). FigTree v.1.4 (Rambaut 2012) was used to visualise the tree. Distance matrices (*p*-distances) were calculated using MEGA v.12.

## Results

The mosquito specimens (53 females, four males and three larvae) were morphologically identified as *Anopheles maculipennis* s. l. The females had well-marked spots on the wings formed by dark scales and the males had only two parabasal setae on the gonocoxite (not three, as males of *An. claviger*).

**Barcoding, phylogeny and distribution of anopheline mosquitoes**

**Figure 3.**
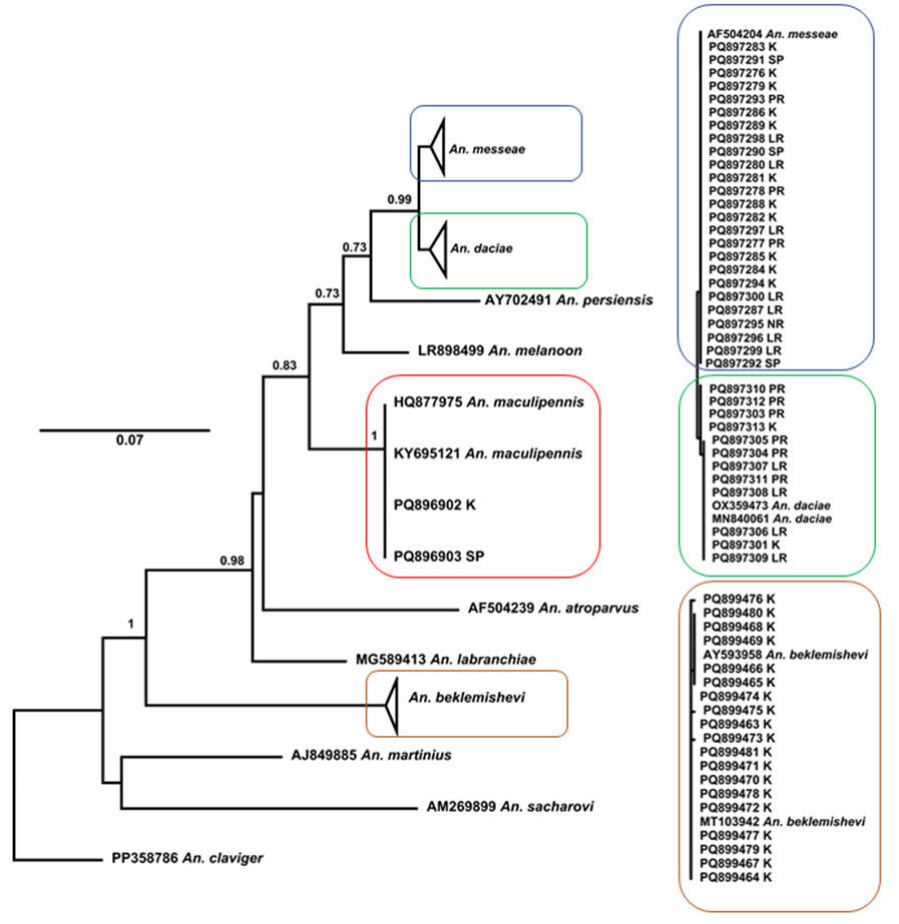
**Maximum Likelihood analysis of the ITS2 region of Maculipennis group using the K2+G+I model**. Maximum Likelihood bootstrap values, based on 1000 replicates, are indicated at the nodes. The scale bar indicates the expected number of substitutions per site. Designations: *K* – Karelia, *SP* – St. Petersburg, *LR* – Leningrad Region, *NR* – Novgorod Region, *PR* – Pskov Region.

In accordance with the phylogenetic analysis, all newly-obtained sequences were grouped into well-supported clades with conspecific reference sequences of *Anopheles beklemishevi* (17F and 2L), *An. maculipennis* s. str. (1F, 1M), *An. daciae* (11F, 1M) and *An. messeae* (22F, 2M and 1L) (Fig. 3). In Karelia, most of the 31 specimens of *An. maculipennis* s. l. belonged to *An. beklemishevi* (17F and 2L), nine specimens of *An. messeae* (8F, 1M), two specimens of *An. daciae* (1F, 1M) and one male of *An. maculipennis*. In Leningrad Region, *An. messeae* (10F, 1L), *An. daciae* (4F) and *An. maculipennis* s. str. (1F) were identified. In Pskov Region, *An. daciae* (6F) and *An. messeae* (3F, 1M) were identified. The only female collected in Novgorod Region belonged to *An. messeae*.

*Anopheles beklemishevi*. Newly-obtained sequences of *An. beklemishevi* grouped into one clade with specimens of this species from Siberia (AY593958, Kampen (2005) and HE659700, Vaulin and Novikov (2012), GenBank, unpublished) and specimens from northern Finland (MT103942, Culverwell et al. (2021)). The total variability of the sampling of *An. beklemishevi* specimens for the partial ITS2 region was 0.6%. Genetic differences within the samples had different values, which were probably largely due to the small amount of material. For instance, both specimens from Munozero Village (Fig. 1; locality 2) were identical, while three sequences of mosquitoes from Paanajärvi differed from each other by 0.18%. P-distances of mosquito sequences from Lososinnoe Village were 0.46%. The sequences of mosquitoes from Gomselga Village were the most diverse (p-distances = 0.87%). Considering differences between *An. beklemishevi* populations, the mosquitoes from Gomselga Village and from Munozero Village were the most different from each other (p-distance 0.84 %), followed by those from Gomselga Village and from Lososinnoe Village (p-distance 0.73 %). The mosquito specimens from Paanajärvi differed less than from the other populations: p-distances were 0.63% for Munozero, 0.58% for Gomselga and only 0.36% for Lososinnoe.

*Anopheles maculipennis* s. str. The two newly-obtained sequences formed a well-supported clade with specimens of *An. maculipennis*: HQ877975 from Turkey (Simsek et al. 2011) and KY695121 from Finland (Culverwell 2018). The sequences were identical even though they were obtained from geographically distant collection localities.

**Figure 4.**
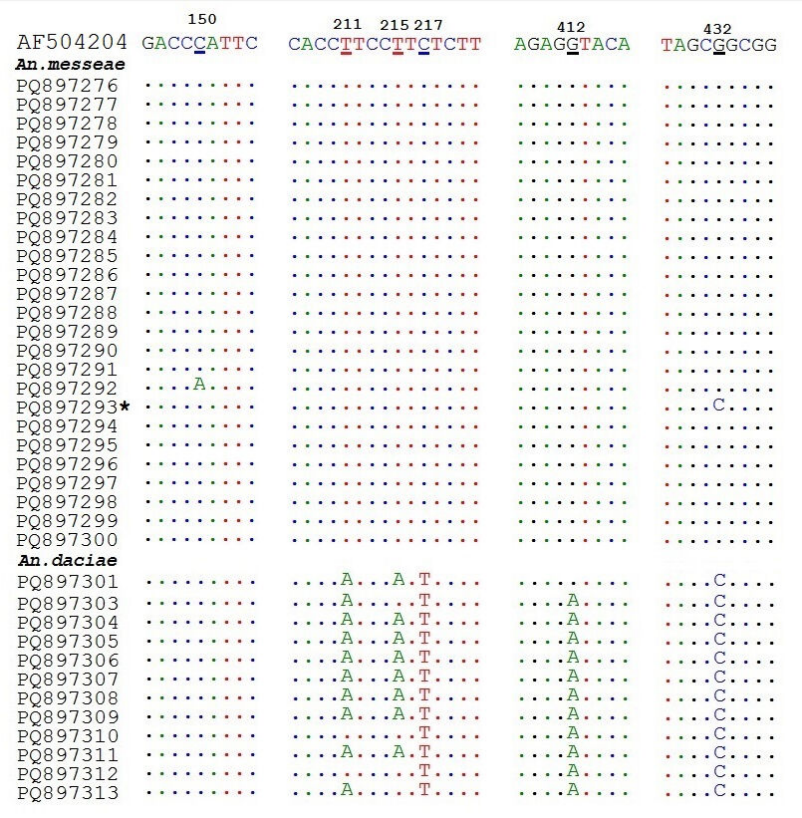
**Variations in five diagnostic nucleotides of ITS2 sequences in *Anopheles messeae* and *Anopheles daciae***. The first line is a sequence of *Anopheles messeae* AF504204 (Linton et al. 2005). Nucleotides corresponding to the reference ones are shown as dots, others are shown as letters. The sequence of specimen considered as a hybrid Anopheles messeae × daciae is marked by an asterisk.

*Anopheles daciae* / *An. messeae*. Twelve specimens of *An. daciae* and 25 specimens of *An. messeae* were identified, based on molecular data (Fig. 3). These two species have a widely overlapping geographical distribution and are mostly recognised, based on ITS2 sequence data only. The representations of nucleotide variants in positions 150, 215, 217, 412 and 432 of ITS2 sequences (Fig. 4), which are considered as diagnostic nucleotides for *An. messeae* and *An. daciae* (Nicolescu et al. 2007; Naumenko et al. 2020; Lilja et al. 2020; Calzolari et al. 2021; Culverwell et al. 2021; Brusentsov et al. 2023), were also revealed.

The number of variations was greater in *Anopheles daciae* than in *An. messeae* (Fig. 4). Four haplotypes were identified with various combinations of substitutions at the diagnostic nucleotides. Most of the *An. daciae* specimens (8) had combination CAATAC. Four specimens showed different patterns in two diagnostic positions: 215 or 217. It is noteworthy that the sequence of mosquito PQ897301 has a transition at position 412 resulting in a combination GC, which indicates that it may be a hybrid. The same was revealed in some previous studies (i.e. Naumenko et al. (2020); Calzolari et al. (2021); Brusentsov et al. (2023)).

There were two haplotypes amongst sequences of the ITS2 region of *Anopheles messeae* regardless of the collection locality. The most numerous was CTTCGG. One *An. messeae* specimen (PQ897292) representing the second haplotype had A at position 150. Another specimen (PQ897293), which could be considered as a hybrid, had С at position 432, which is characteristic of *An. daciae* (Fig. 4). The p-distances between *An. messeae* and *An. daciae* specimens in Karelia was 0.8-1.0%.

**Morphology of female head**

A total of 39 female *Anopheles maculipennis* s. l. specimens (13 *An. beklemishevi*, 16 *An. messeae* and 10 *An. daciae*) out of the 49 females with measured head structures were included in the statistical analysis. We excluded nine damaged specimens in which not all the measurements could be made properly as well as the single specimen of *An. maculipennis* s. str. (the calculation of discriminant functions is possible only if several specimens are present).

**Figure 5.**
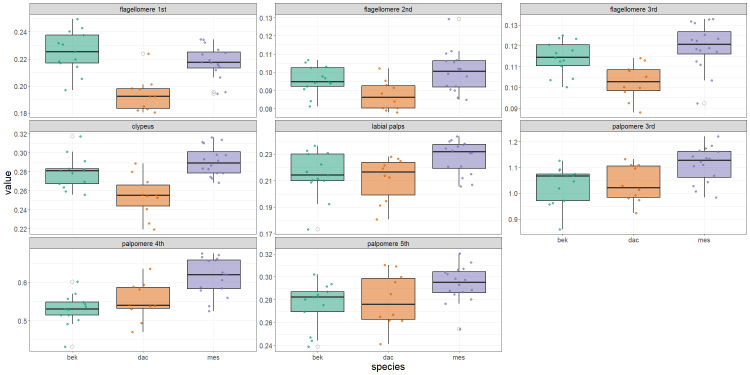
Variation in eight morphological characters of the female head in *Anopheles beklemishevi*, *Anopheles messeae* and *Anopheles daciae*. The meaning of each mosquito specimen is represented by a coloured dot; the variation of values in each species is displayed using a boxplot, where the box corresponds to the interquartile range (IQR), the whiskers extend to 1.5*IQR from the box edges and open circles beyond the whiskers indicate outliers.

**Figure 6.**
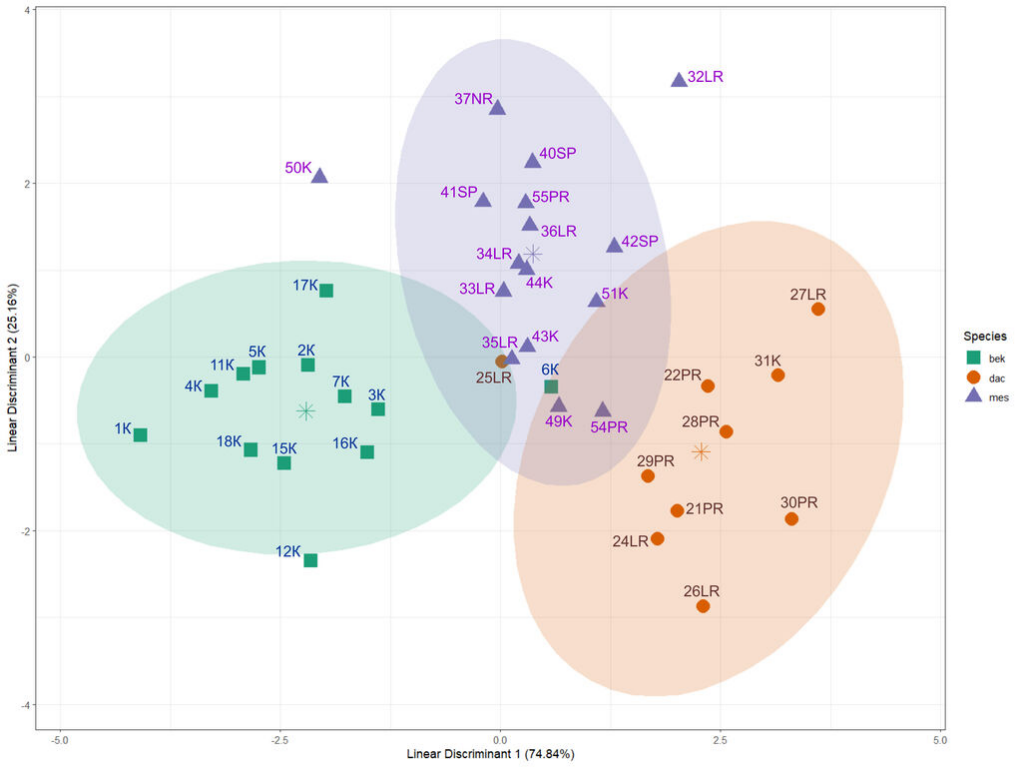
**Visualisation of LDA results for the differentiation of *Anopheles beklemishevi* (bek), *Anopheles messeae* (mes) and *Anopheles daciae* (dac), based on morphological characters of the female head**. Points represent specimens coloured according to the species, asterisks indicate centroids and ellipses show 95% confidence intervals for each species. Axes show the first two linear discriminants: LD1 (explains 74.8% of the variance) and LD2 (explains 25.2% of the variance).

Though the chosen characteristics overlapped to a large degree (Fig. 5), MANOVA showed that the species of mosquito had a statistically significant influence on the complex of morphological characters (Wilks' Lambda = 0.112, F = 7.22, p < 0.001). LDA, based on eight morphological characters of the head, effectively separated the species (Fig. 6). After 10-fold cross-validation, the model achieved an accuracy of 82%, while its accuracy on the full dataset was 92%. There were only three minor classification discrepancies regarding three specimens: one female of *Anopheles daciae* (25LR) erroneously attributed to An. *messeae*, one female of *An. messeae* (54PR) erroneously attributed to *An. daciae* and one female of *An. beklemishevi* (6K) erroneously identified as *An. messeae* (Fig. 6). The first linear discriminant function (LD1) explained 74.8% of the variance and was mainly determined by characters related to the length of flagellomeres (1^st^, 2^nd^ and 3^rd^ segments) (Table 1). The second linear discriminant function (LD2), which explained the remaining 25.2% of the variance, makes an additional contribution to the separation of the species, on the basis of the length of the clypeus, flagellomeres of the 3^rd^ segment and palpomeres of the 5^th^ segment.

Discriminant analysis optimisation with backward stepwise selections made it possible to reduce the set of characters considerably without impairing classification efficiency (classification accuracy was about 90%). To identify the species, we suggest using the equation (below) obtained by backward selections employing only two characters: length of 1^st^ flagellomere and length of 4^th^ palpomere.

LDA generates calculated characters (Fisher’s linear discriminant functions) that characterise each object in the group. A specific equation has the form:

Z = A + a_1_*x_1_ + a_2_*x_2_ +...+ a_m_*x_m_,

where a_1_, a_2_ and a_m_ are the coefficients indicating the contribution of each character to the clasification capability of function. The total number of functions to be calculated equals the number of groups under study (Korosov and Gorbach 2017). In our case, there are three of them: *Anopheles daciae*, *An. messeae* and *An. beklemishevi*. Using these equations, we can estimate the value of Z for each mosquito individual and assign that individual to a certain group (species) depending on the result.

Having studied eight morphological characters, we suggest focusing on just two on them (length of 1^st^ flagellomere and length of 4^th^ palpomere), which contribute the most to differentiation between the three *Anopheles* species. Exclusion of other characters from the analysis does not compromise the quality of the discriminant function.

The assignment of an *Anopheles* specimen to one of the three species can be determined by the highest calculated value:

*Anopheles beklemishevi*: -159.4 – 8.82 *pal4th* + 1420.5*fl1st*

*Anopheles daciae*: -119.4 + 67.35 *pal4th* + 1033.6 *fl1st*

*Anopheles messeae*: -150.9 + 71.94 *pal4th* + 1174.3 *fl1st*

## Discussion

Molecular and morphological results of our study offer some new insights into the distribution of the Maculipennis group. Though our data do not ascertain the northern range boundaries of *Anopheles maculipennis* s. str., *An. daciae*, *An. messeae* and *An. beklemishevi* (see Suppl. material 1), they supplement the data on their overall distribution. For instance, *An. daciae* was found in Leningrad Region and Pskov Region for the first time. Previously, this species was recorded in Finland and Russia (St. Petersburg and Arkhangelsk Region). Its presence in Karelia was confirmed by molecular data for the first time. Our results support the suggestion of Moskaev et al. (2024) that the distribution boundary of *An. daciae* passes south of the 64^th^ parallel.

Analysis of ITS2 sequences of our specimens of *Anopheles daciae* showed that they were represented in NWR by four haplotypes. Two of them were found in Karelia (two localities) and three in Pskov Region (two localities). The most widespread haplotype of *An. daciae* was found in the north-eastern Leningrad Region and in the southern Pskov Region, the collection localities being more than 500 km apart. Similar variation in the ITS2 of *An. daciae* was also reported elsewhere across its distribution: in Finland, Sweden, Poland, Germany, Italy, Latvia and various parts of Russia (Lilja et al. 2020; Calzolari et al. 2021; Culverwell et al. 2021; Brusentsov et al. 2023).

Most of our specimens of *Anopheles* were identified by ITS2 as *An. messeae*. They were recorded at all collection localities, except the northernmost one (Paanajärvi). The record of this species at Munozero Village roughly corresponds in latitude to the northernmost one in Finland (Culverwell et al. (2021), see Suppl. material 1). DNA sequences of *An. messeae* collected at different localities in our study did not differ from those (PP115571, PP115572) collected in northern Karelia (Kem and Loukhi Districts) by Moskaev et al. (2024). Besides, most of our specimens of *An. messeae* (except two specimens differing by one nucleotide substitution) were identical by ITS2 to many specimens of this species from various parts of its range, from England, Finland and Sweden to Greece and across the entire Russia up to the easternmost location in Yakutsk (Lilja et al. 2020; Calzolari et al. 2021; Culverwell et al. 2021; Ibáñez-Justicia et al. 2022; Brusentsov et al. 2023). This means that genetic diversity of *An. messeae* is much lower than that of *An. claviger*, which had many more haplotypes both in southern Karelia and across its range in general (Khalin et al. 2024).

A similar situation was observed for *Anopheles maculipennis* s. str. Two specimens from collection localities more than 300 km apart were completely identical by ITS2 to each other and to many specimens from distant parts of the range, including HQ877975 from Turkey (Simsek et al. 2011) and KY695121 from Finland (Culverwell 2018). Moreover, ITS2 sequences of *An. maculipennis* s. str. from Karelia were identical not only to those from other European countries such as Italy, Poland, Belgium, the Netherlands and Croatia (Marinucci et al. 1999; Bušić et al. 2021; Ibáñez-Justicia et al. 2022; Schaffner et al. 2024), but also to those from Iran (Asadi Saatlou et al. 2019).

*Anopheles beklemishevi* was found at four localities in Karelia, including one near the Arctic Circle (Fig. 1, point 1). This is one of the northernmost records of *An. beklemishevi* in Russia and confirmed by ITS2 data. Taking into account that *An. beklemishevi* was recorded in Finland beyond the Arctic Circle (Culverwell et al. 2021) and in north-eastern Karelia (Moskaev et al. 2024), we can assume that there is a stable population of this species in northern Karelia.

Since *Anopheles beklemishevi* is widely distributed the northern Palaearctic, it was advisable to compare genetic differences between populations from several northern localities. As expected, the lowest p-distance value = 0.05% for ITS2 site was between mosquitoes from Kuusamo and Kainuu in Finland (Culverwell et al. 2021) and Paanajärvi, because these regions are very close (Fig. 1, Suppl. material 1). The difference with the regions further south was more significant, making up 0.2% with mosquitoes from Lososinnoe Village, 0.4% from Munozero Village and 0.5% from Gomselga Village. The difference between the p-distances of ITS2 sequences of *An. beklemishevi* collected in NWR and Siberian specimens AY593958 (Kampen 2005) and HE659700 (Vaulin and Novikov 2012) was 0.45%.

During the examination of Maculipennis group, some morphological differences between females, males and larvae of different species were observed. For instance, adults differ in the wing geometry (Bellin et al. 2021; Demipci 2021; Calzolari et al. 2024) and in the shape of wing scales on certain veins (Darsie and Samanidou-Voysdjoglou 1997) and male terminalia (Martini 1933; Shute 1935; Denisova 1940; Shabanova and Sibataev 2007), while larvae differ in the chaetotaxy of abdominal segments IV and V (Bates 1939; Suzzoni-Blatger and Sevin 1982) or the prothorax (Sibataev and Shabanova 2007) and pupae differ in the same of abdominal segments VI and VII (Sicart and Ruffie 1960). However, these characters vary widely and overlap in different species. Furthermore, species within *Anopheles maculipennis* s. l. are sometimes identified, based on egg morphology, for example, the presence of floats and egg surface colouration (Becker et al. 2020).

Morphological characters of the female head were used to identify *Anopheles* species by Gutsevich (1973b), but no differences were found between *An. maculipennis* s. str. and *An. messeae* (*An. daciae* and *An. beklemishevi* were described later). In our work, ITS2-based species identification is complemented by the morphological study of the female head. We have demonstrated that, although the length of palpomeres and flagellomeres as well as the clypeus and labial palps varied widely and partially overlapped in *An. daciae*, *An. messeae* and *An. beklemishevi* (Fig. 5), these species diverged quite distinctly in a set of characters when applying multivariable calculus (Fig. 6). LDA, as a multidimensional scaling technique, is a standard method in zoological research. We have previously used LDA to differentiate not only between species (Lebedeva et al. 2025), but also between hybrids (Bugmyrin et al. 2015).

Our results highlight the complexity of the taxonomic characters of *Anopheles maculipennis* s. l., shown in previous investigations (Calzolari et al. 2021; Smitz et al. 2021; Calzolari et al. 2024). However, our morphological data of the female head and ITS2 sequences provide some indications that *An. daciae* and *An. messeae* may be distinct species. In our study, specimens of both species with different haplotypes, as well as one hybrid *An. daciae* / *An. messeae* (Fig. 4), were close to the centroids of species groups based on the set of morphological characters. Conversely, morphologically distinct individuals of *Anopheles daciae* and *An. messeae* (Fig. 6, points 25LR and 54PR) had ITS2 sequences characteristic of their species, without any substitutions. Nevertheless, our morphological analysis indicates that the characters of female head of *An. maculipennis* s. l. may be promising for species identification. The discriminant equations obtained in our study may be useful for a preliminary identification of *An. daciae*, *An. messeae* and *An. beklemishevi*, based on morphological characters, which is to be followed by the final identification, based on ITS2 sequences.

## Conclusions

The data available to date indicate that five *Anopheles* species occur in NWR: *An. claviger*, *An. beklemishevi*, *An. daciae*, *An. messeae* and *An. maculipennis* s. str. (Perevozkin et al. 2012; Khalin et al. 2021a; Khalin et al. 2024; Moskaev et al. 2024; present study). Their distribution has been confirmed by ITS2 data. *Anopheles plumbeus* and *An. atroparvus*, known from Estonia, Belarus and Kaliningrad Region of Russia (Suslo 2020; Khalin et al. 2021a; Kirik et al. 2022; Suslo and Khalin 2025), have not been recorded in NWR yet. New records of *An. daciae* in Leningrad and Pskov Regions made it possible to update its range, while the records of other members of *An. maculipennis* s. l. in Karelia confirm the literature data, based on chromosome identification (see, Stegniy (1991); Perevozkin et al. (2012); Moskaev et al. (2024)). The specimens of *An. beklemishevi* and *An. daciae* collected in our study were fairly diverse in the ITS2 sequence. A similarly high diversity of this sequence is also characteristic of *An. claviger* (Khalin et al. 2024). However, all specimens of *An. maculipennis* s. str. and almost all specimens of *An. messeae* examined in our study were identical, based on ITS2. Our study of the morphology of the female head was based on a small number of specimens sampled only in the northern part of the ranges of *An. daciae*, *An. messeae* and *An. beklemishevi* and its results require additional research on a greater amount of material. Future research may finally result in complete morphological descriptions of the species of the Maculipennis group.

## Supplementary Material

2291AE41-6204-57DC-A207-0AEDCD97FBEA 10.3897/BDJ.13.e164756.suppl1 Supplementary material 1 Northernmost records of anopheline mosquitoes in Fennoscandia and north-western Russia, according to the collection material (circles) and the literature data (triangles) Data type Image (map) Brief description Map of Fennoscandia and north-western Russia.
Designations: blue circles and triangles – *Anopheles beklemishevi*; the same magenta – *An. messeae*; the same green – *An. daciae*; the same brown – *An. maculipennis* s. str. The triangles with the orange border show the literature data up to 1990. File: oo_1369651.jpg https://binary.pensoft.net/file/1369651 Sergey V. Aibulatov, Daria I. Lebedeva, Alexei V. Khalin, Natalia A. Lyutikova, Daniil D. Fedorov, Liubov A. Bespyatova, Alexei V. Polevoi, Gelena A. Lunina, and Sergey V. Bugmyrin

BDE4A682-687E-585B-893E-CD788D5DAAEE 10.3897/BDJ.13.e164756.suppl2 Supplementary material 2 Biotopes of collection locations Data type Image (photo) Brief description Collection localites 1 (*A*), 3 (*B*, *C*), 10 (*D*), 11 (*E*) and 12 (*F*) (see Mosquito sampling). File: oo_1369652.jpg https://binary.pensoft.net/file/1369652 Sergey V. Aibulatov, Daria I. Lebedeva, Alexei V. Khalin, Natalia A. Lyutikova, Daniil D. Fedorov, Liubov A. Bespyatova, Alexei V. Polevoi, Gelena A. Lunina, and Sergey V. Bugmyrin

C8432AB7-C14A-5A16-BAF1-97FEA94377A6 10.3897/BDJ.13.e164756.suppl3 Supplementary material 3 Material examined Data type Table Brief description Stages, collection localities and Genbank Accession Numbers of *Anopheles* specimens.
Designations: *K* – Karelia, *SP* – Saint-Petersburg, *LR* – Leningrad Region, *NR* – Novgorod Region, *PR* – Pskov Region. *F* – female, *M* – male, *L* – larva, *L/M* – larva reared in an adult male.
The number in the first column corresponds to specimen number on Fig. 6. File: oo_1369663.docx https://binary.pensoft.net/file/1369663 Sergey V. Aibulatov, Daria I. Lebedeva, Alexei V. Khalin, Natalia A. Lyutikova, Daniil D. Fedorov, Liubov A. Bespyatova, Alexei V. Polevoi, Gelena A. Lunina, and Sergey V. Bugmyrin

## Figures and Tables

**Table 1.** LDA coefficients showing the contribution of each morphological character to the differentiation of *Anopheles beklemishevi*, *Anopheles messeae* and *Anopheles daciae*, based on linear discriminants LD1 and LD2.

**Morphological characters**	**LD1**	**LD2**
flagellomere 1^st^	-91.8	-17.7
flagellomere 2^nd^	71.9	7.9
flagellomere 3^rd^	-87.2	33.0
palpomere 3^rd^	6.3	-8.1
palpomere 4^th^	16.9	24.9
palpomere 5^th^	18.8	-27.0
clypeus	-27.0	39.4
labial palps	-0.7	-0.2
Explained variance	74.8%	25.2%
