# Supplementary figures and images for: Anopheline mosquitoes of north-western Russia (Diptera, Culicidae): updated distribution and morphological characters
Source: Biodivers Data J. 2025 Aug 27;13:e164756. doi: 10.3897/BDJ.13.e164756 (PMC12409332; doi:10.3897/BDJ.13.e164756)

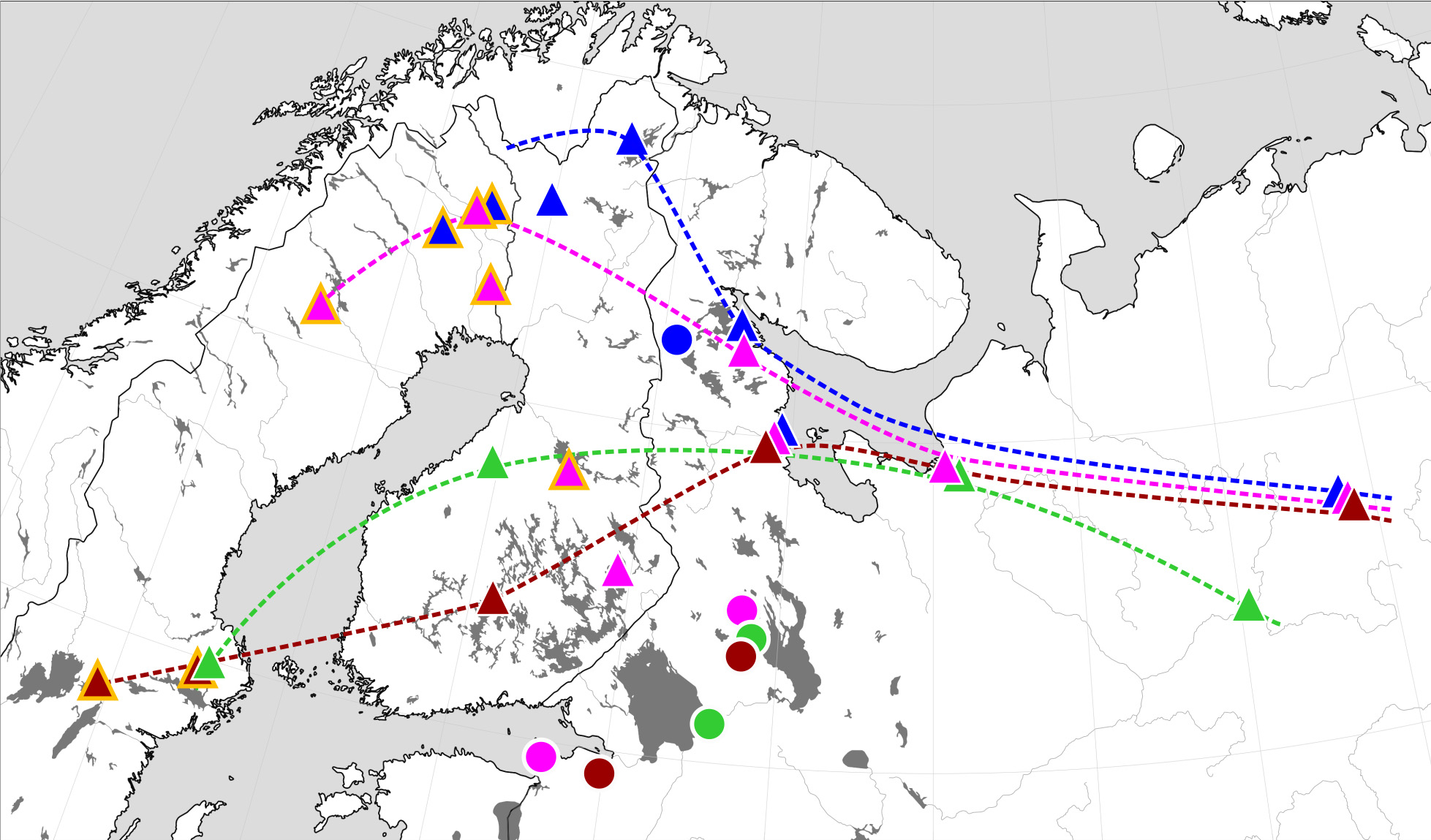

Supplement: Supplementary material 1 — Northernmost records of anopheline mosquitoes in Fennoscandia and north-western Russia, according to the collection material (circles) and the literature data (triangles) [file bdj-13-e164756-s001.jpg]

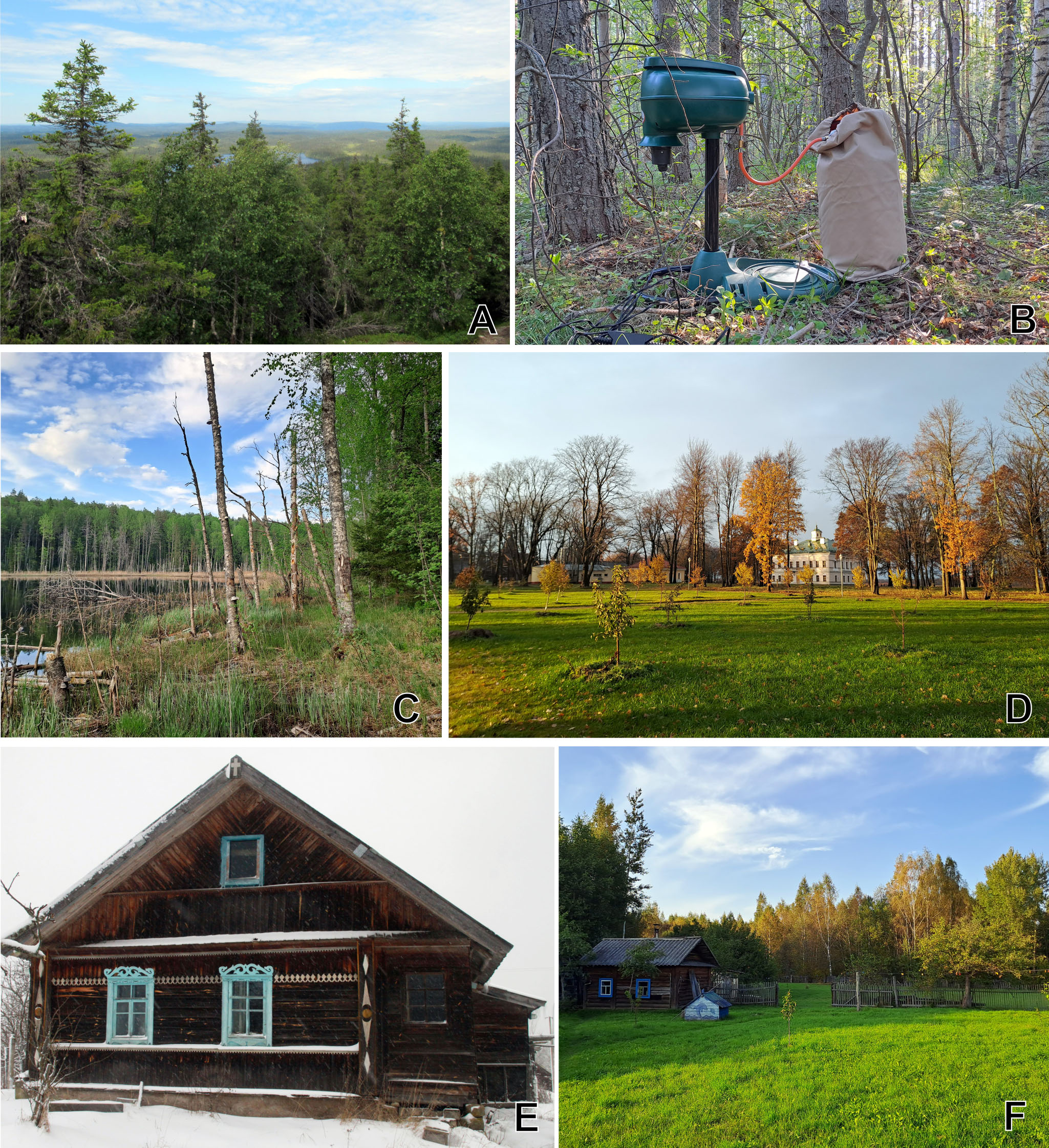

Supplement: Supplementary material 2 — Biotopes of collection locations [file bdj-13-e164756-s002.jpg]
