# Supplementary material for: Anopheline mosquitoes of north-western Russia (Diptera, Culicidae): updated distribution and morphological characters
Source: Biodivers Data J. 2025 Aug 27;13:e164756. doi: 10.3897/BDJ.13.e164756 (PMC12409332; doi:10.3897/BDJ.13.e164756)
Supplement: Supplementary material 3 — Material examined [file bdj-13-e164756-s003.docx]

**Supplementary material**

for online publication only

Sergey V. Aibulatov, Daria I. Lebedeva, Alexei V. Khalin*, Natalia A. Lyutikova, Daniil D. Fedorov, Liubov A. Bespyatova, Alexei V. Polevoi, Gelena A. Lunina, and Sergey V. Bugmyrin

**Table S3**. Material examined: stages, collection localities and Genbank Accession Numbers of *Anopheles* specimens

| **No** | **Species** | **Stage** | **Number of collection locality** | **Region** | **Genbank Accession Number ITS2** |
| --- | --- | --- | --- | --- | --- |
| 1 | *An. beklemishevi* | F | 5 | K | PQ899481 |
| 2 | *An. beklemishevi* | F | 5 | K | PQ899480 |
| 3 | *An. beklemishevi* | F | 5 | K | PQ899479 |
| 4 | *An. beklemishevi* | F | 5 | K | PQ899478 |
| 5 | *An. beklemishevi* | F | 5 | K | PQ899477 |
| 6 | *An. beklemishevi* | F | 5 | K | PQ899476 |
| 7 | *An. beklemishevi* | F | 5 | K | PQ899475 |
| 8 | *An. beklemishevi* | F | 5 | K | PQ899474 |
| 9 | *An. beklemishevi* | L | 3 | K | PQ899473 |
| 10 | *An. beklemishevi* | F | 1 | K | PQ899472 |
| 11 | *An. beklemishevi* | F | 1 | K | PQ899471 |
| 12 | *An. beklemishevi* | F | 1 | K | PQ899470 |
| 13 | *An. beklemishevi* | F | 2 | K | PQ899469 |
| 14 | *An. beklemishevi* | F | 2 | K | PQ899468 |
| 15 | *An. beklemishevi* | F | 3 | K | PQ899467 |
| 16 | *An. beklemishevi* | F | 3 | K | PQ899466 |
| 17 | *An. beklemishevi* | F | 3 | K | PQ899465 |
| 18 | *An. beklemishevi* | F | 3 | K | PQ899464 |
| 19 | *An. beklemishevi* | L | 3 | K | PQ899463 |
| 20 | *An. daciae* | M | 4 | K | PQ897313 |
| 21 | *An. daciae* | F | 12 | PR | PQ897312 |
| 22 | *An. daciae* | F | 12 | PR | PQ897311 |
| 23 | *An. daciae* | F | 11 | PR | PQ897310 |
| 24 | *An. daciae* | F | 6 | LR | PQ897309 |
| 25 | *An. daciae* | F | 6 | LR | PQ897308 |
| 26 | *An. daciae* | F | 6 | LR | PQ897307 |
| 27 | *An. daciae* | F | 6 | LR | PQ897306 |
| 28 | *An. daciae* | F | 11 | PR | PQ897305 |
| 29 | *An. daciae* | F | 11 | PR | PQ897304 |
| 30 | *An. daciae* | F | 11 | PR | PQ897303 |
| 31 | *An. daciae** | F | 5 | K | PQ897301 |
| 32 | *An. messeae* | F | 9 | LR | PQ897300 |
| 33 | *An. messeae* | F | 9 | LR | PQ897299 |
| 34 | *An. messeae* | F | 9 | LR | PQ897298 |
| 35 | *An. messeae* | F | 9 | LR | PQ897297 |
| 36 | *An. messeae* | F | 9 | LR | PQ897296 |
| 37 | *An. messeae* | F | 10 | NR | PQ897295 |
| 38 | *An. messeae* | M | 4 | K | PQ897294 |
| 39 | *An. messeae* | L/M | 12 | PR | PQ897293 |
| 40 | *An. messeae* | F | 7 | SP | PQ897292 |
| 41 | *An. messeae* | F | 7 | SP | PQ897291 |
| 42 | *An. messeae* | F | 8 | SP | PQ897290 |
| 43 | *An. messeae* | F | 5 | K | PQ897289 |
| 44 | *An. messeae* | F | 5 | K | PQ897288 |
| 45 | *An. messeae* | L | 6 | LR | PQ897287 |
| 46 | *An. messeae* | F | 2 | K | PQ897286 |
| 47 | *An. messeae* | F | 2 | K | PQ897285 |
| 48 | *An. messeae* | F | 2 | K | PQ897284 |
| 49 | *An. messeae* | F | 3 | K | PQ897283 |
| 50 | *An. messeae* | F | 3 | K | PQ897282 |
| 51 | *An. messeae* | F | 3 | K | PQ897281 |
| 52 | *An. messeae* | F | 6 | LR | PQ897280 |
| 53 | *An. messeae* | F | 6 | LR | PQ897279 |
| 54 | *An. messeae* | F | 11 | PR | PQ897278 |
| 55 | *An. messeae* | F | 11 | PR | PQ897277 |
| 56 | *An. messeae* | F | 12 | PR | PQ897276 |
| 57 | *An. maculipennis* | F | 8 | SP | PQ896903 |
| 58 | *An. maculipennis* | M | 4 | K | PQ896902 |

Designations: K – Karelia, SP – Saint-Petersburg, LR – Leningrad Region, NR – Novgorod Region, PR – Pskov Region. F – female, M – male, L – larva, L/M – larva reared in an adult male. No corresponds to specimen number on the Fig. 6.
